# Skin Tone Representation of Early Lyme Disease in Medical Education Resources: Gaps and Implications for Equity

**DOI:** 10.1177/23821205251407778

**Published:** 2026-01-14

**Authors:** Nathaniel Baffoe-Mensah, Jules B. Lipoff, Christine M. Forke

**Affiliations:** 1 Penn Medicine Lancaster General Hospital, 4399 Family Health Residency Program, Lancaster, PA, USA; 2 Master of Public Health Program, Perelman School of Medicine at the University of Pennsylvania, Philadelphia, PA, USA; 3 Department of Dermatology, Lewis Katz School of Medicine, 12314 Temple University, Philadelphia, PA, USA; 4 Department of Family Medicine and Community Health, 14640 Perelman School of Medicine at the University of Pennsylvania, Philadelphia, PA, USA; 5 Center for Public Health, Perelman School of Medicine at the University of Pennsylvania, Philadelphia, PA, USA; 6 Leonard Davis Institute of Health Economics, University of Pennsylvania, Philadelphia, PA, USA

**Keywords:** Lyme disease, erythema migrans, race, racial disparities, health inequities, medical education, dermatology, educational resources

## Abstract

**Background:**

Cutaneous Lyme disease presents differently in light versus dark skin, and delayed diagnosis can increase outcome severity. Insufficient exposure to manifestations of Lyme disease in dark skin during medical training may contribute to health inequities due to late or missed diagnoses. It remains unclear how Lyme disease, specifically, is represented in commonly used medical training materials. To inform curricula updates, we identified primary educational resources used for teaching dermatology at top-tier U.S. medical schools and assessed the representation of erythema migrans on light and dark skin in these materials.

**Methods:**

In this cross-sectional content analysis, commonly used training resources for 50 top U.S. medical schools were identified by reviewing websites and syllabi and contacting schools when information was unavailable. Resource images were categorized as “light-skinned” or “dark-skinned” using the Fitzpatrick scale (I-III vs IV-VI). Proportions and counts of light-skinned and dark-skinned images were compared to U.S. demographics, resource format (print-/web-based), and age of publication (pre-/post-2020).

**Results:**

Sixteen resources, containing 47 erythema migrans images, were identified. Two of 16 (12.5%) resources included dark-skinned images; both were web-based resources. None of the print resources or those published before 2020 included dark-skinned images. The proportions of light-skinned (n = 44, 93.6%) and dark-skinned (n = 3, 6.4%) images were significantly different from U.S. demographics (*p* = .03).

**Conclusions:**

Among commonly used medical student resources, few contain images of erythema migrans on dark skin; these were only found in web-based resources published since 2020. This differential representation has the potential to contribute to inequitable diagnosis and treatment across racial groups.

## Introduction

Increasing attention is being paid to the role that racial bias in medical education plays in perpetuating healthcare inequities.^1^ Despite recent emphasis on diversity, equity, and inclusion, under-representation of disease presentation in specific racial groups in textbooks, lectures, case studies, and clinical training materials undermines these efforts.^2,3^

It is well-recognized that some dermatological infectious diseases are disproportionately visually depicted in certain races over others and that light skin is most commonly used for images in medical textbooks^4–8^; however, it is less clear if these resources are commonly used during training. Presence or absence of representation of certain racial groups in medical training materials informs the associations doctors make between race and disease risk.^9^ When groups are under-represented in training materials or are represented only in conjunction with certain topics, they could be erroneously perceived to have a lower or higher prevalence of certain diseases, potentially resulting in improper diagnoses and treatments.^2^ Some dermatological conditions, like skin cancer or keloids, are more prevalent in light or dark skin, respectively^10–12^; in these cases, imbalanced imagery reflects disease prevalence. However, many dermatological conditions affect all racial groups, with clinical presentation differing across skin tones.^13^ Lack of exposure to diverse visual examples during training limits development and consolidation of clinical acumen, which can limit clinicians’ ability to recognize clinical signs of disease more broadly, thereby contributing to diagnostic inaccuracies and delayed care.^3^

Exposure to visual markers of disease progression across skin tones becomes especially salient in the context of Lyme disease, where accurate recognition is essential for early diagnosis. Lyme disease, one infectious dermatological disease suspected of being under-represented on dark skin in training materials,^4^ is the leading cause of vector-borne bacterial disease in humans worldwide and the most common tick-borne disease in the United States.^14^ Erythema migrans, a pathognomonic rash occurring in about 70% of cases, is a key clinical feature.^14^ If recognized and treated early, debilitating sequelae can be avoided. Epidemiological studies of Lyme disease, however, report delays in diagnosis among patients with darker skin tones versus lighter skin tones; patients with lighter skin tones are 2.8 times more likely to be diagnosed with early-stage disease.^9^ Furthermore, among patients with darker skin, considerably more diagnoses are made at later stages of disease.^9,15^ These differences are particularly important because late-stage Lyme disease often presents with arthritis and Bell's palsy, and symptoms may persist for months to years despite treatment with recommended antibiotic therapy and can substantially impact quality of life.^16,17^

Prior studies have documented under-representation of darker skin tones broadly in general dermatology training materials,^2^^4–8^ but studies examining visual imagery for Lyme disease in training materials are limited. Because Lyme disease affects all populations but presents differently across skin tones, it serves as an exemplar case to highlight how gaps in curricular resources may create inequities in recognition and clinical outcomes. It is crucial that trainees are exposed to the range of disease presentations across skin tones, yet studies have not yet quantitatively analyzed how erythema migrans is visually depicted in medical student resources that are relied upon for training. This knowledge gap is critical, given well-documented disparities in Lyme disease outcomes as a result of delays in diagnosis.^9,15,18^ Therefore, the objective of our study was to examine the most commonly used resources recommended to trainees at top U.S. medical schools, including textbooks across various medical specialties and web-based platforms, to evaluate whether erythema migrans imagery equally represents light and dark skin tones.

## Materials and Methods

This is a cross-sectional descriptive study using content analysis of available images in medical training resources. The reporting of this study conforms to the Strengthening the Reporting of Observational Studies in Epidemiology (STROBE) statement^19^ (Supplemental File 1).

### Sample Selection

Recommendations for resources were obtained from 2 primary sources: (a) the top 50 highest-ranked medical schools in the United States, based on the 2022 U.S. News & World Report^20^ and (b) the top-rated resource lists section of “First Aid USMLE Step 1 2021”^21^ and “First Aid USMLE Step 2 CK 10th edition.”^22^

Data were collected between March 2022 and September 2024. Information regarding school-specific resource recommendations was directly retrieved from medical school websites, typically listed under the “education” or “curriculum” sections. Course-specific text recommendations were available through online course descriptions, library guides, or downloadable Portable Document Format (PDF) files detailing each course and its prerequisite texts. Multiple email attempts were made to contact schools lacking readily available recommendations online. This work was exempted from Institutional Board Review, as human subjects were not involved.

### Inclusion and Exclusion Criteria

**Figure 1.**
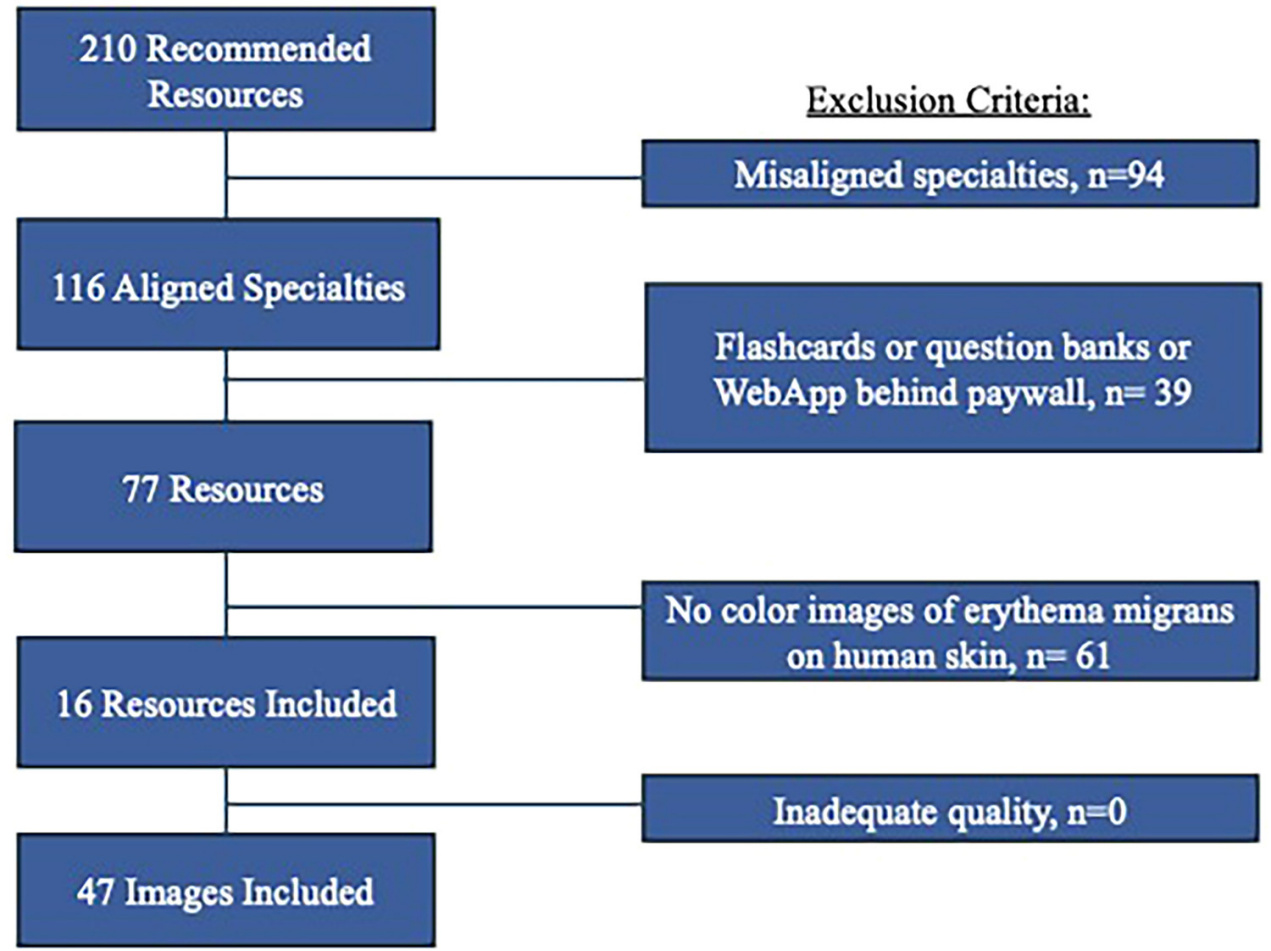
Inclusion and exclusion criteria for resources and images depicting erythema migrans on human skin.

Commonly recommended resources—medical textbooks, websites, and web applications—pertaining to the clinical practice of dermatology, pathology, microbiology, infectious disease, family medicine, emergency medicine, internal medicine, and pediatrics were included (Figure 1). These specialties were chosen due to their relevance and likelihood of discussing Lyme disease and its early-stage manifestations. Resources focused on topics such as anatomy, embryology, behavioral science, biochemistry, cell biology, histology, immunology, pharmacology, physiology, psychiatry, neurology, OB/GYN, and surgery were excluded. Other excluded resources were: subscription-based online question banks, mobile apps, and flashcard apps, as well as any other resources behind paywalls because their databases could not be directly accessed for review.

For images, additional inclusion and exclusion criteria were based on approaches used in prior studies investigating racial diversity through image analysis.^23,24^ For resources to be included in this study, recommended medical textbooks and online medical resources had to contain at least one *color* image depicting early-stage Lyme disease on human skin.^24,25^ Images were excluded if the quality was insufficient to accurately determine the skin tone (Figure 1). For each included resource, we documented: specialty choice of the subject matter; title; year of publication or last update; format (digital or physical); and the count of each image type included (light-skinned, dark-skinned, or both).

### Image Classification

Two reviewers identified all pictures referencing early-stage Lyme disease in the available resources and independently coded the skin tone depicted in each image using the 6-point Fitzpatrick scale.^26^ All images were analyzed using a standardized approach.^24,27,28^ Images rated as I-III on the Fitzpatrick scale were categorized as light-skinned, while those rated as types IV-VI were categorized as dark-skinned.^24,25^ For comparison to U.S. Census data for race, individuals who self-identified as Black, Native American, non-White Hispanic, Asian/Pacific Islander, and multiracial individuals were classified as dark-skinned. Inter-rater reliability between reviewers was assessed using Cohen's kappa (*k*) coefficient with 30 randomly selected images of different skin tones.^29^

### Statistical Analysis

Proportions of light-skinned and dark-skinned images across all resources were compared using a Chi-square goodness of fit test to determine if our observed sample distribution reflected the expected 60% to 40% racial distribution of light-skinned and dark-skinned individuals in the United States based on the 2020 Census data.^30^ The types of images (light-skinned, dark-skinned, or both) in each resource were compared by the kind of resource (i.e., medical textbook or web-based application) and publication/update year using a Fisher's exact test. Image counts (for total, light-skinned, dark-skinned, both) were compared by the kind of resource and publication/update year using the Wilcoxon rank sum test, with median and first and third quartiles (Q1 and Q3) reported. Alpha was set to 0.05 for all analyses. The unit of analysis was based on the identified training resources and images; thus, sample size reflected the number of qualifying resources identified through school-recommended materials. While not powered a priori for inferential testing due to the sample being determined organically, a limited set of a priori exploratory hypotheses were theoretically derived and guided all analyses; as a result, no statistical adjustments for multiple testing were applied.^31^ Publication year was grouped as pre- versus post-2020 to capture potential changes associated with curricular updates and diversity initiatives. Resource type was grouped as textbook versus web-based to reflect the two primary modalities of medical student learning materials widely accessible across institutions. There were no missing data points for key variables, as all included images were of sufficient quality for skin tone assessment.

## Results

Of the 50 top medical school-affiliated websites reviewed, 28 (56%) had information regarding recommended texts available on their website. The remaining 22 (44%) either required school-specific login information to access course information (n = 3/22, 13.6%) or did not have student textbooks or resource recommendations on their website (n = 19/22, 86.4%). After multiple requests for information from these 22 schools, 5 (22.7%) responded; however, no additional resources were identified from these communications.

Overall, 210 resources were initially identified. Ninety-four (44.8%) were excluded due to misaligned specialties, and 39 (18.6%) were excluded due to inaccessibility behind paywalls (e.g., books, flashcards, and question banks), leaving 77 (36.7%) resources for analysis. Of these, 16 (20.8%) included color images of erythema migrans on human skin, and all images (n = 47, 100%) in these resources were of sufficient quality to assess skin tone (Figure 1). In terms of reviewers’ categorization of images based on the Fitzpatrick scale, the prevalence-adjusted kappa value was 1.0 (perfect agreement).

### Image Type as the Unit of Analysis

Table 1 shows characteristics of the 16 medical student textbooks and resources. Half (8 of 16) were published or updated prior to 2020, and half were published during or after 2020 (Table 2). Of the 16 recommended resources, most (14 of 16, 87.5%) depicted images of erythema migrans in only light-skinned patients. All resources containing dark-skinned images were published since 2020, but differences in the types of images included in the resources by publication year were not statistically significant (*p* = .47)—likely due to being underpowered. In contrast, compared to textbooks, resources that were web-based were more likely to contain images of erythema migrans in dark-skinned patients, with 2 of the 3 (66.7%) web-based resources having dark-skinned images, compared to none of the 13 (0%) print resources (*p* = .03). Among these two online resources with images on dark skin, the ratio of light-skinned to dark-skinned images was 7:2 and 8:1.

**Table 1.** Characteristics of Erythema Migrans Images in Recommended Resources Used by Top U.S. Medical Schools.

Recommended resources from medical schools	Latest update/Publication year	Resource	Image type	Total images	Light-skinned images^a^	Dark-skinned images^b^
Schaechter's Mechanisms of Microbial Disease, 5th Ed	2013	Textbook	Only light-skinned	1	1	0
Crush Step 1: The Ultimate USMLE Step 1 Review	2017	Textbook	Only light-skinned	2	2	0
USMLE Step 1 Secrets in Color	2017	Textbook	Only light-skinned	1	1	0
Fitzpatrick's Color Atlas and Synopsis of Clinical Dermatology, 8th Edition	2017	Textbook	Only light-skinned	2	2	0
Rapid Review: Pathology	2018	Textbook	Only light-skinned	1	1	0
Medical Microbiology: A Guide to Microbial Infections by Michael Barer	2018	Textbook	Only light-skinned	1	1	0
First Aid	2019	Textbook	Only light-skinned	1	1	0
Mim's Medical Microbiology	2019	Textbook	Only light-skinned	1	1	0
Warren Levinson (Review of Medical Microbiology)	2020	Textbook	Only light-skinned	1	1	0
Mandell, Douglas, and Bennett's Principles and Practice of Infectious Diseases	2020	Textbook	Only light-skinned	3	3	0
UpToDate	2020	Online Web App	Only light-skinned	8	8	0
CDC	2020	Online Website	Both light-skinned & dark-skinned	9	7	2
Dermatology Secrets (Whitney A. High)	2021	Textbook	Only light-skinned	1	1	0
Medical Microbiology by Murray et al	2022	Textbook	Only light-skinned	5	5	0
Sherris and Ryan's Medical Microbiology	2022	Textbook	Only light-skinned	1	1	0
AMBOSS	2022	Online Web App	Both light-skinned & dark-skinned	9	8	1

a Fitzpatrick Scale I-III.

b Fitzpatrick Scale IV-VI.

**Table 2.** Description of the Types of Images Found in Each Kind of Resource and by Publication Date.

	Resource		Publication date	
	Textbooks	Web-based app	Before 2020	Since 2020
*Resources as the unit of analysis (n* *=* *16)*	*(n* *=* *13, 81%)*	*(n* *=* *3, 19%)*	*(n* *=* *8, 50%)*	*(n* *=* *8, 50%)*
Resources with…				
Only light-skinned images	13 (100%)	1 (33%)	8 (100%)	6 (75%)
Only dark-skinned images	0 (0%)	0 (0%)	0 (0%)	0 (0%)
Both White and dark-skinned images	0 (0%)	2 (66%)	0 (0%)	2 (25%)
*Images as the unit of analysis (n* *=* *47)*	*(n* *=* *21, 45%)*	*(n* *=* *26, 55%)*	*(n* *=* *10, 21%)*	*(n* *=* *37, 79%)*
Images in each resource that are…				
Light-skinned images	21 (100%)	23 (88%)	10 (100%)	34 (92%)
Dark-skinned images	0 (0%)	3 (12%)	0 (0%)	3 (8%)

### Image Count as the Unit of Analysis

Across all 16 resources, 47 images were identified and analyzed; 44 images (93.6%) were on light skin tones, and 3 images (6.4%) were on dark skin tones. Resources published or updated since 2020 contained nearly 4 times more images (n = 37, median = 3.5, Q1 = 1, Q3 = 7) compared to those from before 2020 (n = 10, median = 1.0, Q1 = 1, Q3 = 1.5), although this difference was not statistically significant at our predefined alpha of 0.05 (*p* = .06). The median number of images in web-based resources was higher than for textbooks (web-based median = 9, Q1 = 5, Q3 = 9 vs textbook median = 1, Q1 = 1, Q3 = 2; *p* = .005), as was the median number of dark-skinned images (web-based median = 1, Q1 = 0, Q3 = 2 vs textbook median = 0, Q1 = 0, Q3 = 0; *p* = .002). Compared to the 2016 racial demographic distribution within the United States, with 61.3% of the population identifying in racial groups with light skin tones and 39.7% otherwise,^30^ there were fewer images with dark skin tones observed in medical student resources than expected (*p* = .03).

## Discussion

Our results suggest that commonly used U.S. medical student resources do not adequately represent erythema migrans. Resources include few images overall and do not reflect diversity across skin tones, as they default to representation on lighter skin tones. Particularly because Lyme disease has less severe outcomes when identified and treated early, discrepancies with visual representation in training materials could delay identification for those with darker skin tones, leading to racial inequities in care.^3,15,16,18^ Interestingly, web-based resources include more images of erythema migrans on darker skin tones than traditional textbooks, possibly because of more space for images, but the ratio of light-skinned to dark-skinned representation in both sources is highly imbalanced (overall 44 light vs 3 dark). The low number of erythema migrans images (n = 47) identified across all training resources is troubling, particularly since nearly all resources reviewed had only one image on lighter skin tones. In total, these findings reflect a need to increase trainees’ exposure to erythema migrans at various stages of disease and across all skin tones.

Our study showed that the imbalance of images for erythema migrans found in commonly used training materials is similar to findings reported by researchers who examined other common dermatological conditions on darker skin tones in general nursing and medical textbooks. For instance, a study focused on the widely used *Bates’ Guide to Physical Examination and History Taking* found a significant under-representation of darker skin tones in images depicting general dermatological conditions.^2^ Another study that analyzed several prominent U.S. medical textbooks, including dermatology-specific texts, had similar conclusions that indicate a pervasive lack of diversity across skin tones.^32,33^ Expanding on that work, a recent study focusing specifically on Lyme disease showed that medical students struggle to diagnose erythema migrans on darker skin.^34^ Coupled with our results, these findings show that medical students have limited exposure to visual imagery of disease presentation on different skin tones for broad and specific dermatological diseases, which may contribute to under-recognition and perpetuate inequities in care.

Demographics of the U.S. are expected to shift significantly, with the combined populations of Hispanics, African Americans, Asian/Pacific Islanders, and American Indian/Alaskan Natives increasing from 39% to 44% by 2030 and to 56% by 2060.^30^ Given these shifts, it's crucial that medical training materials reflect the full range of disease presentations across all skin tones. Under-representation of erythema migrans on darker skin in educational resources is commonly cited as one reason for missed and delayed diagnoses of Lyme disease in patients identifying as Black.^3,15,18,35^ Under-representation limits trainees’ exposure to racial and ethnic differences in disease presentation, potentially affecting future clinicians’ and machine learning models’ ability to recognize disease in under-represented groups. Inequities in identifying erythema migrans early can promote diagnostic bias, inaccurate treatment plans, and subpar clinical outcomes that hinge on race, especially since untreated Lyme disease can result in more severe outcomes. Studies on visual representation in medical resources before 2020 have asserted that there has been limited progress towards equitable imagery,^2,27,36^ but a recent study specifically focusing on online resources for rheumatological conditions found improvements in representation of darker skin tones in online materials since 2020.^37^

Despite being underpowered to detect the small differences we found in the number of dark-skinned images published before and since 2020, we also saw clear patterns emerge showing meaningful improvements—albeit far from what is needed. Positive patterns were evident in the number of *resources* that included dark-skinned images, which increased from 0 to 2, and in the number of dark-skinned *images* present, which increased from 0 to 3. Notably, even though there were far more print resources, all of the dark-skinned images were from web-based resources, suggesting more electronic resources were being used since 2020. However, with only 3 dark-skinned images published since 2020, considerable room for improvement remains. This persistent under-representation, despite heightened awareness of racial equity, highlights the slow pace of systemic change and reinforces the need for intentional efforts to diversify visual content in clinical training. Future work should continue to monitor whether these positive trends continue, particularly as more online resources are utilized.

Practicality has been suggested as one reason that more images of erythema migrans are presented on lighter skin tones. For example, publishers experience pressure to limit pages and images due to print costs, especially in hardcopy materials.^38^ Nine of 13 (69.2%) textbooks in our study included only 1 image, and only 2 (15.3%) books had 3 or more images. If publishers are limited to 1 or 2 images, they are likely to choose images where the rash is more visually distinct—a condition which favors lighter skin tones. This supports increasing the use of web-based resources in education, as they have more images overall and more diversity of images across skin tones. Second, while the hallmark rash may indeed be easier to recognize on lighter skin, that increases the need for trainees to be exposed to a wider variety of images, especially presentations that may seem atypical, to build strong diagnostic competence across all skin tones and scenarios. Finally, many educational resources are designed to align with standardized exams, which may further promote similar biases. If board exams assess competence using primarily lighter-skinned imagery, the resources created to prepare students for these exams will likely reflect the same imbalance. Therefore, an important future step could include quantifying the diversity of images and questions within USMLE content to indicate if a top-down approach to the issue may be warranted, as addressing this issue at the level of exam content may be a key lever for driving broader change in clinical education materials.

Of note, almost half of all resources in our study (n = 7, 44%) and approximately one-third of textbooks (n = 4, 31%) included multiple images. Four textbooks included between 2 and 5 color images, yet none contained images of the rash on darker skin tones, despite it presenting differently in this group.^2^ Because of the difficulty identifying early Lyme on darker skin tones, more visual aids within educational materials are warranted to display various representations and stages of disease in this group. With decreased awareness of Lyme disease and symptoms among those identifying as Black, more representative visual imagery online could also aid public health messaging campaigns for patients that might facilitate earlier diagnosis.^18^

We found that the online resources recommended to medical students, which can be edited more quickly and easily, were the only resources to contain images on darker skin tones. Even though online resources contained many more images compared to textbooks and had the capacity to include high-quality color prints, the ratio of light-skinned to dark-skinned images was highly unbalanced.

The insights gained in this study can be useful for guiding medical educators and website developers who are working to minimize racial inequities in healthcare. Not only is digital space considerably less expensive than hard copy print materials,^39^ but online images also allow for higher resolution and the use of full-color images, which can offer more accurate representations of rashes on darker skin tones compared to what can be conveyed through black-and-white textbook photos. Educators should be intentional about integrating the use of web-based resources into their curricula to show diverse pathology and disease presentations, thereby ensuring future clinicians will be adequately equipped to diagnose and treat an increasingly racially and ethnically diverse U.S. population. Further, as medical schools address health inequities, they may want to consider subscribing to web services and explore if those behind paywalls might allow their students to access more diverse images for training. Medical schools may also consider creating online, institutional, crowd-shared resources allowing students and faculty to share images from their own clinical experiences. These findings also can serve as a call to website and content developers at CDC, AMBOSS, and UpToDate to add a broader range of images to their sites that depict dermatological conditions on darker skin tones at various stages of the disease, just as many of the resources do for lighter skin tones. Nolen^3^ notes that online sites such as *Brown Skin Matters* and *VisualDx* are building repositories of images showing manifestations of various skin conditions to address these inequities. Solutions like these can expand opportunities for trainees and practicing clinicians alike to have a more balanced representation of how erythema migrans and Lyme disease present across various skin tones.

Limitations must be noted. Our sample was limited to medical student textbooks and web-based resources and did not include lecture materials or other supplemental educational materials being used by students such as question banks or mobile and flash card applications. While resources we studied can be used by all clinicians throughout their careers, these resources were identified specifically regarding medical student training; we cannot make inferences about their use beyond medical school. Classification of skin tone as light-skinned or dark-skinned is based on previously established methodology,^24,27,28,36^ but binary classification does not fully capture the true diversity of various race subcategories. Furthermore, some studies use V-VI Fitzpatrick classification to represent dark skin; we classified dark skin more broadly using classifications IV-VI, which if anything should have biased our results to detect fewer differences in representation, thus making our findings even more compelling. Additionally, this investigation was limited to schools with curricula details and resources readily available online and may not fully capture all recommended resources. However, we found considerable overlap across universities in the resources recommended, and schools without their curriculum identified online were scattered throughout the ranks of the top schools, not limited to schools at the top or bottom of the rankings, giving more confidence in our findings. Also, when these schools were queried by email, all relevant resources they provided were already captured in our list. If resources we could not access (e.g., paywalled question banks or mobile applications) had distributions that were similar to those analyzed, any bias would be small; if they contained a higher proportion of dark-skin images, our estimates of under-representation would be overstated. However, the latter scenario is unlikely given the similarities we found across recommended resources and prior studies consistently documenting under-representation.

## Conclusion

This study assessed the visual representation of early-stage Lyme disease in recommended medical student resources. Our findings demonstrate a disparity in the visual depiction of erythema migrans, the hallmark sign of Lyme disease, within commonly recommended textbooks and web-based resources. Although online platforms included more images and broader representation across skin tones, images of the rash on dark skin tones were sparse, emphasizing a critical gap in medical education. This lack of diversity contradicts shifting U.S. demographics and may contribute to diagnostic bias and disparate clinical outcomes. To overcome this discrepancy, partners in medical education must prioritize diverse and inclusive visuals in training materials leveraging full-color, high-resolution images that can better depict the rash at various stages of progression on various skin tones. With concerted collective efforts, future medical providers can be better equipped to provide effective and equitable patient care in an increasingly diverse society.

## Supplemental Material

sj-doc-1-mde-10.1177_23821205251407778 - Supplemental material for Skin Tone Representation of Early Lyme Disease in Medical Education Resources: Gaps and Implications for Equity Supplemental material, sj-doc-1-mde-10.1177_23821205251407778 for Skin Tone Representation of Early Lyme Disease in Medical Education Resources: Gaps and Implications for Equity by Nathaniel Baffoe-Mensah, Jules B. Lipoff and Christine M. Forke in Journal of Medical Education and Curricular Development
